# Radiation-Induced Lymphopenia Risks of Photon Versus Proton Therapy for Esophageal Cancer Patients

**DOI:** 10.14338/IJPT-20-00086

**Published:** 2021-04-07

**Authors:** Saba Ebrahimi, Gino Lim, Amy Liu, Steven H. Lin, Susannah G. Ellsworth, Clemens Grassberger, Radhe Mohan, Wenhua Cao

**Affiliations:** 1 Department of Industrial Engineering, University of Houston, Houston, TX, USA; 2 Department of Radiation Physics, The University of Texas MD Anderson Cancer Center, Houston, TX, USA; 3 Department of Radiation Oncology, The University of Texas MD Anderson Cancer Center, Houston, TX, USA; 4 Department of Radiation Oncology, Indiana University, Indianapolis, IN, USA; 5 Departments of Radiation Oncology, Massachusetts General Hospital, Boston, MA, USA

**Keywords:** IMRT, PSPT, IMPT, lymphopenia

## Abstract

**Purpose:**

To assess possible differences in radiation-induced lymphocyte depletion for esophageal cancer patients being treated with the following 3 treatment modalities: intensity-modulated radiation therapy (IMRT), passive scattering proton therapy (PSPT), and intensity-modulated proton therapy (IMPT).

**Methods and Materials:**

We used 2 prediction models to estimate lymphocyte depletion based on dose distributions. Model I used a piecewise linear relationship between lymphocyte survival and voxel-by-voxel dose. Model II assumes that lymphocytes deplete exponentially as a function of total delivered dose. The models can be fitted using the weekly absolute lymphocyte counts measurements collected throughout treatment. We randomly selected 45 esophageal cancer patients treated with IMRT, PSPT, or IMPT at our institution (15 per modality) to demonstrate the fitness of the 2 models. A different group of 10 esophageal cancer patients who had received PSPT were included in this study of in silico simulations of multiple modalities. One IMRT and one IMPT plan were created, using our standards of practice for each modality, as competing plans to the existing PSPT plan for each patient. We fitted the models by PSPT plans used in treatment and predicted absolute lymphocyte counts for IMRT and IMPT plans.

**Results:**

Model validation on each modality group of patients showed good agreement between measured and predicted absolute lymphocyte counts nadirs with mean squared errors from 0.003 to 0.023 among the modalities and models. In the simulation study of IMRT and IMPT on the 10 PSPT patients, the average predicted absolute lymphocyte count (ALC) nadirs were 0.27, 0.35, and 0.37 K/μL after IMRT, PSPT, and IMPT treatments using Model I, respectively, and 0.14, 0.22, and 0.33 K/μL using Model II.

**Conclusions:**

Proton plans carried a lower predicted risk of lymphopenia after the treatment course than did photon plans. Moreover, IMPT plans outperformed PSPT in terms of predicted lymphocyte preservation.

## Introduction

Radiation-induced lymphopenia (RIL), lymphocyte depletion, is a common toxicity of radiation therapy and is associated with worse outcomes in a number of solid tumors, including esophageal cancer [1–4]. Because lymphocytes have a substantial role in the body's anticancer immune response, severe lymphopenia can reduce patients' survival even in the early stages of tumor progression [4–8]. Multiple recent studies have shown that severe lymphopenia is strongly associated with poor treatment outcomes for patients with cervical [8, 9], pancreatic [10, 11], rectal [12], lung [5, 13], and esophageal [2, 14] cancers. Thus, preservation of the lymphocytes from radiation damage is crucial for the effectiveness of radiation therapy, and it is critical to understand the clinical and dosimetric factors affecting the severity and incidence of RIL and develop strategies for its mitigation.

Radiation-induced lymphopenia occurs presumably owing to the high radiosensitivity of lymphocytes. In conventional photon radiation therapy, the large low- and medium-dose bath expose substantial fractions of circulating lymphocytes. Clinical data show that dose distribution patterns and fractionation regimens significantly influence lymphocyte depletion [15, 16]. Dose distribution patterns from protons and photons can differ greatly, and the dosimetric advantages of the state-of-the-art proton therapy over photon therapy in terms of sparing of organs at risk and normal tissue have been demonstrated extensively [17, 18]. Furthermore, intensity-modulated proton therapy (IMPT) performs further better than intensity-modulated radiation therapy (IMRT) in terms of normal tissue sparing [19].

RIL risk likely depends on the treatment modality. Recent studies have reported greater lymphocyte depletion in patients treated with photon therapy than with proton therapy [2, 4, 13, 14, 20]. For example, Shiraishi et al [2] reported that proton beam therapy was associated with a lower risk of grade 4 lymphopenia compared with IMRT in esophageal cancer patients receiving neoadjuvant chemoradiotherapy.

In this study, we aimed to analyze probability of severe lymphopenia induced from radiation therapy using simple models of lymphocyte loss based on dose distribution patterns. Such models have the potential to help physicians identify patients at high risk of grade 4 lymphopenia based on the evaluation of dose distributions of treatment plans. In this regard, we modeled and compared expected absolute lymphocyte count (ALC) depletion kinetics in esophageal cancer patients treated with the following 3 different modalities: IMRT, passive-scattering proton therapy (PSPT), and IMPT.

## Materials and Methods

In this section, we will describe 2 prediction models for lymphocyte depletion based on radiation doses and then report the patient selection for model validation as well as treatment planning of IMRT and IMPT for patients treated with PSPT.

### ALC Depletion Prediction Using a Piecewise Linear Lymphocyte Survival Function

On the basis of the dose distributions in each patient, we can estimate the ALC during treatment by using a piecewise linear relationship between lymphocyte survival and dose per fraction for each modality. A piecewise linear function can be modeled by interpolating previous findings about radiation-induced lymphocyte death [21, 22]. Nakamura et al [22] reported the percentages of surviving lymphocytes as 90%, 50%, 10%, and 0% for radiation doses of 0.5 Gy, 2 Gy, 3 Gy, and 6 Gy or higher for each fraction, respectively. This estimation assumes that all circulating lymphocytes may receive dose by the end of treatment (after 28 fractions in this study). The lymphocyte survival probability for each voxel *i*, *S_i_*, after receiving a fractional dose *d* (in Gy), can be calculated using the following piecewise linear function:

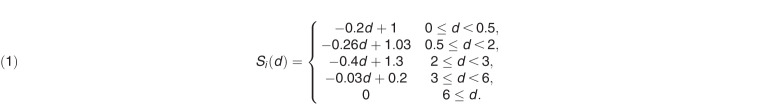



We assume that the initial number of lymphocytes in 1 μL of body volume can be estimated by multiplying the pretreatment ALC value for each patient by the percentage of blood in 1 μL of body volume. We also assume that all fast and slowly circulating lymphocytes are distributed uniformly throughout the irradiated volume and receive dose during radiation therapy treatment. The average percentage of blood in the human body is 7% of body weight/volume [23], so the number of lymphocytes in 1 μL of body volume before treatment can be estimated as *L*_0_ *=* ALC_0_ *×* 0.07 (cells ×1000/μL). Therefore, the total number of lymphocytes in a given patient's body would be

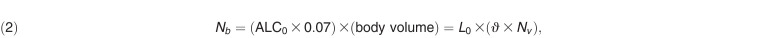
where *ϑ* is the volume of each voxel and *N_v_* is the total number of voxels in the body for each patient.

The total number of lymphocytes remaining in the body volume after 1 fraction is calculated by summing the number of surviving lymphocytes in all voxels as follows:

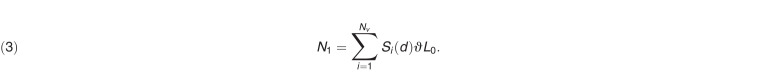



To find the ALC value after the entire course of treatments, the probability of lymphocyte survival after delivering *k* fractions (*P_k_*) is then calculated using the ratio of the remaining lymphocytes to the initial number of lymphocytes in the body.

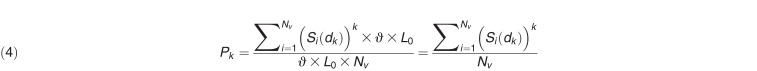



Thus, the expected final value of ALC after *k* fractions (*k ≥* 1) can be estimated using the following equation:

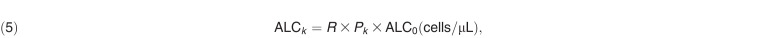
where *R* is the fitting parameter and ALC_0_ is the ALC value before treatment. *R* can be determined by fitting the model to ALC measurements collected from patients. It is also used to account for all other compounding contributing factors, such as tumor location and histology, treatment volume, chemo status, and so on.

Note that only weekly ALC measurements could be available in current clinical practice, rather than measurement per fraction. Therefore, there would be at most 5 or 6 weekly ALC data points available for patients receiving a conventional photon or proton treatment, such as in this study.

### ALC Prediction Using Exponential Curve Fitting

Previous studies have shown that ALC loss follows exponential decay in setting of total body radiation in primates [24] and in humans (accidental exposure) [25]. An exponential fitting method was also used to study ALC loss in partial body radiation therapy [26]. As an alternative to the piecewise linear model described above, we can also model patient ALC using an exponential function of accumulated delivered dose. Specifically, the measured weekly ALC data points from RT patients can be used to fit an exponential function based on the total delivered dose to the body (ie, the sum of doses in all voxels in treatment field) after *k* fractions (*D_k_* > 0) as

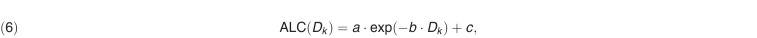
where *a* is a fixed parameter indicating the initial ALC before starting the treatment ALC_0_, *b* is an index of an individual patient's lymphocytes' sensitivity to dose, and *c* is added to the exponential function to account for the replenishment of lymphocytes after irradiation. To avoid a negative value of ALC, nonnegative constraint was placed on c (ie, *c* ≥ 0).

Using the weekly ALC data points and delivered dose in each week, we can determine the fitted values of the *b* and *c* parameters for each patient. In the planning study, we used the same parameters to predict ALC during treatment for PSPT, IMRT, and IMPT plans according to individual patient's ALC baseline value (*a*) and the delivered dose values of *D_k_*.

In addition, one could use the ALC measurement data from only the initial weeks of treatment (eg, 3 weeks) to fit the exponential ALC function of dose to predict the final ALC after the entire treatment course. The rationale for this approach was to determine whether patient-specific factors, including lymphocyte radiation sensitivity, derived from initial treatment fractions, is predictive of loss of lymphocytes by the end of treatment.

### Patient Selection and Treatment Planning

The model validation study and the in silico simulation study of retrospective patient cohorts were approved by the institutional review board of The University of Texas MD Anderson Cancer Center. Forty-five esophageal cancer patients, who had been treated with IMRT, PSPT, or IMPT (15 per modality) with the same treatment prescription of delivering 50.4 Gy in 28 fractions, were randomly selected to validate the prediction models of lymphocyte depletion. Dose distributions used in their treatments and weekly ALC measurements throughout the treatment course (including baseline ALC before treatment) were collected from our database. All patients had at least 5 ALC data points.

Ten other esophageal cancer patients treated with PSPT (50.4 Gy delivered in 28 fractions) were included in the modality comparison study. Five patients had 6 weekly measurements; 4 patients had 5 measurements; and 1 patient had only 4 measurements. ALC nadir was defined as the lowest value among the weekly measurements for each patient. The average ALC nadir for the 10 patients was 0.34 K/μL, ranging from 0.07 to 0.68 K/μL. Grade 4 lymphopenia (G4L) and grade 3 lymphopenia (G3L), according to the Common Toxicity Criteria for Adverse Events, version 5.0 (National Cancer Institute, Bethesda, MD), are defined as ALC < 200 cells/μL and ALC < 500 cells/μL, respectively. Three patients had grade 2 lymphopenia, 4 had grade 3, and 3 had grade 4. All patients received concurrent chemoradiation therapy, during which chemotherapy regimens were doublets of a taxane, fluorouracil, or platinum-based compound. Examples of the PSPT dose distributions for all patients can be found in **Supplemental Figure S1**.

We used MatRad [27], a research-oriented treatment planning system, to create IMRT and IMPT plans for each patient. The prescription dose to the clinical target volume was 50.4 Gy in 28 fractions for all patients. Six or seven beams were used for IMRT plans for these 10 patients based on our planning protocol in clinic. Two or 3 beams were used for IMPT plans with the same beam angles as those used for patients' actual PSPT treatments. In optimization of the IMRT and IMPT plans, the same dosimetric criteria were used for each patient, but objective weights and constraints were adjusted, when necessary, to achieve the best possible target coverage and normal tissue sparing. For optimization and evaluation of PSPT and IMPT plans, we used a constant relative biological effectiveness of 1.1 [28]. All plans were normalized to have 95% of the planning target volume receive the prescription dose.

## Results

### Model Validation

Dose distributions and ALC measurements of 15 patients per treatment modality (IMRT, PSPT, or IMPT) were used to validate both piecewise linear and exponential models. Results of ALC predictions are summarized in Table 1. The average mean body doses were 14.44, 7.37, and 6.12 Gy for IMRT, PSPT, and IMPT treatments, respectively. The average ALC nadir for the 15 patients treated with IMRT, PSPT, and IMPT was 0.17, 0.33, and 0.39 K/μL, respectively. The average predicted ALC nadirs after treatment were 0.15, 0.32, and 0.37 K/μL after IMRT, PSPT, and IMPT treatments using the piecewise linear model, respectively, and 0.12, 0.30, and 0.36 K/μL using the exponential model. The mean squared error was 0.005, 0.023, and 0.003 for IMRT, PSPT, and IMPT treatments based on the piecewise linear model, and 0.005, 0.005, and 0.004 based on the exponential model.

**Table 1.** Mean body dose, ALC baseline, real and predicted ALC nadirs, and associated errors for patients treated with IMRT, PSPT, and IMPT.

**RT modality**	**Mean body dose, Gy**	**ALC_0_**	**Real ALC**	**Piecewise linear model**			**Exponential model**		
				**Predicted ALC nadir**	**MSE**	**MAE**	**Predicted ALC nadir**	**MSE**	**MAE**
IMRT	14.44	1.42 ± 0.46	0.17 ± 0.10	0.15 ± 0.09	0.005	0.064	0.12 ± 0.06	0.005	0.053
PSPT	7.37	1.41 ± 0.57	0.33 ± 0.18	0.32 ± 0.15	0.023	0.104	0.30 ± 0.16	0.005	0.057
IMPT	6.12	1.55 ± 0.57	0.39 ± 0.26	0.37 ± 0.24	0.003	0.040	0.36 ± 0.24	0.004	0.058

**Abbreviations:** RT, radiation therapy; ALC, absolute lymphocyte counts; MSE: mean squared error; MAE: mean absolute error; IMRT, intensity-modulated radiation therapy; PSPT, passive scattering proton therapy; IMPT, intensity-modulated proton therapy.

Note: Unit for ALC values is cells × 1000/μL. Values for ALC are presented as mean ± SD deviation.

For each group of patients, ΔALC (baseline − nadir) from real values and predictions were also calculated (**Supplemental Table S1**). The average ΔALC from measurements were 1.25, 1.08, and 0.97 K/μL for IMRT, PSPT, and IMPT treatments, respectively; the average predicted ΔALC were 1.27, 1.09, and 0.98 K/μL from the piecewise linear model, and 1.30, 1.11, and 0.99 K/μL from the exponential model. Both measured and predicted ALC changes indicate patients receiving IMRT had the most lymphocyte reduction during treatment, while IMPT patients had the least lymphocyte reduction.

As the average baseline ALCs from the patient groups were different among RT modalities, we also compared normalized ΔALC (ie, ΔALC/baseline ALC) (**Supplemental Table S2**). The results also showed good agreement between real values and predictions from the 2 models. The mean squared errors for all treatment modalities and prediction models were less than 0.007, and the mean absolute errors were less than 0.067.

### Dosimetric Characteristics of IMRT, PSPT and IMPT Plans

In the planning study of the 10 patients treated with PSPT, we first compared the dose distributions of the PSPT plans used for treatment and the IMRT and IMPT plans generated for this study, in terms of dose-volume metrics (eg, mean body dose, *V*_5_, *V*_10_, etc.). An example of the dose distributions of IMRT, PSPT, and IMPT plans for a patient (patient 5) can be found in the Supplement (**Supplemental Figure S2**). As expected, the radiation dose using the IMPT plan conformed more closely to the planning target volume than did the PSPT and IMRT plans, and the IMRT plan resulted in the largest dose baths to the body.

The mean body doses, averaged among the 10 patients, were 7.46, 4.84, and 3.85 Gy for IMRT, PSPT, and IMPT plans, respectively. The fractions of the body volume that received different doses were consistently higher for photon therapy (IMRT) plans than for proton therapy (IMPT and PSPT) plans, especially at low doses, such as 5 or 10 Gy. For each dose-volume index, IMPT plans outperformed PSPT plans. More detailed comparison of body dose-volume metrics for the 10 patients among the 3 modalities can be found in **Supplemental Figure S3**.

### Lymphocyte Survival Based on Piecewise Linear Function of Voxel Dose

Using the piecewise linear lymphocyte survival function, we estimated ALCs as a function of delivered radiation dose to each voxel in the patient body for IMRT, PSPT, and IMPT plans for each patient. Before the analysis, we determined the fitting parameter, *R* in (5), to be 26%, based on the average of the measured ALC data for the 10 PSPT patients. The same *R* values were used for all following ALC predations.

**Figure 1.**
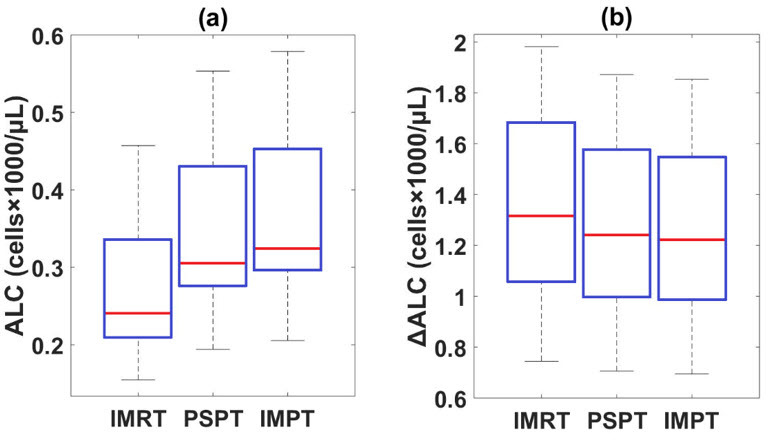
ALC predictions using the piecewise linear method. (A) Box plots indicating estimated ALC after the 3 treatment modalities for 10 esophageal cancer patients. (B) Box plots indicating predicted ALC change before and after the 3 treatment modalities for 10 esophageal cancer patients. Abbreviation: ALC, absolute lymphocyte counts.

The average predicted ALCs after treatment were 0.27 (95% CI [0.21, 0.33]), 0.35 (95% CI [0.27, 0.42]), and 0.37 K/μL (95% CI [0.29, 0.44]) for IMRT, PSPT, and IMPT plans, respectively. **Figure 1A** shows box plots of predicted ALCs at the end of treatment courses for the 3 treatment modalities. **Figure 1B** shows box plots of predicted ALC changes before and after treatment (ΔALC = baseline − nadir). Proton plans showed smaller ALC depletion than did photon plans, and IMPT had the highest ALC nadirs compared with the other 2 plans.

**Figure 2.**
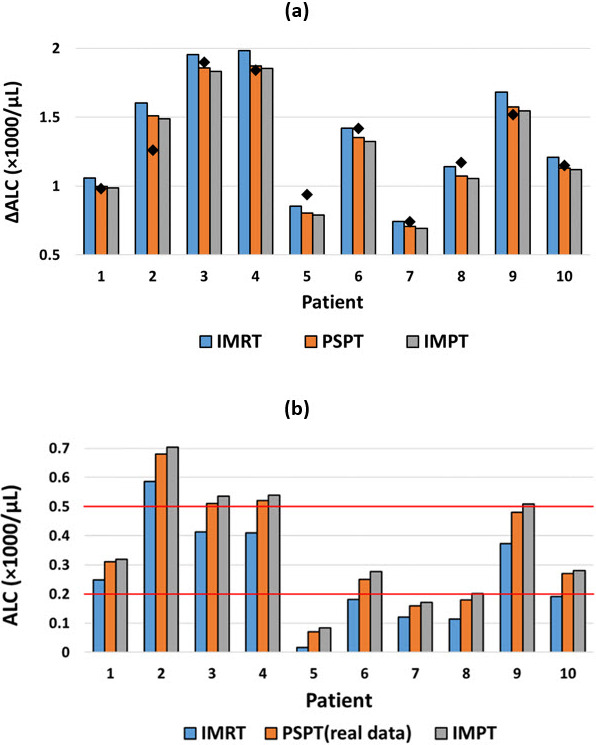
(A) Comparison of predicted ALC change (baseline − nadir) using the piecewise linear method for IMRT, PSPT, and IMPT plans for 10 esophageal cancer patients. The changes in ALC from measured ALC data for PSPT treatments are indicated by the black diamonds. (B) Predicted ALC nadirs for IMRT and IMPT treatments (blue and gray) using the piecewise linear method compared with the measured ALC nadir for PSPT treatments (orange). Abbreviations: ALC, absolute lymphocyte counts; IMPT, intensity-modulated proton therapy; IMRT, intensity-modulated radiation therapy; PSPT, passive scattering proton therapy.

The predicted ALC changes for each of the 10 patients for the 3 modalities are shown in **Figure 2A**. These ΔALC values for IMRT plans were higher than those of IMPT and PSPT plans for all 10 patients and support the hypothesis that protons consistently cause less ALC depletion than do photons. Actual ALC changes from measured ALC data for PSPT treatments are indicated by black diamonds. Of note, we observed that the ALC estimates for PSPT plans were relatively close to the real measured data, with a mean absolute error of 0.075 and a mean squared error of 0.010. It reassures that this simple linear model of lymphocyte survival from radiation dose can provide good predictions.

Recent studies have reported a lower risk of grade 4 lymphopenia for patients treated with proton therapy compared with patients treated with photon therapy [2, 14, 20]. **Figure 2B** shows the predicted ALC nadirs for IMRT and IMPT treatments, which were calculated by subtracting the predicted ALC change from the measured ALC baseline, for each of the 10 patients. **Figure 2B** demonstrates the predicted ALC nadir if each patient had been treated by IMRT or IMPT instead of PSPT. Grade 4 lymphopenia occurred in patients 5, 7, and 8 after PSPT treatment. Based on the predicted ALC nadirs, grade 4 lymphopenia might have been avoided for patient 8 if they had been treated with IMPT instead. Patients 6 and 10 had grade 3 lymphopenia after PSPT; however, they may have developed grade 4 lymphopenia if they had been treated with IMRT. Patients 3 and 4 were predicted to have developed grade 3 lymphopenia if IMRT had been used instead of PSPT. In all cases IMPT would have reduced the risk of RIL.

### Exponential Fitting

**Figure 3.**
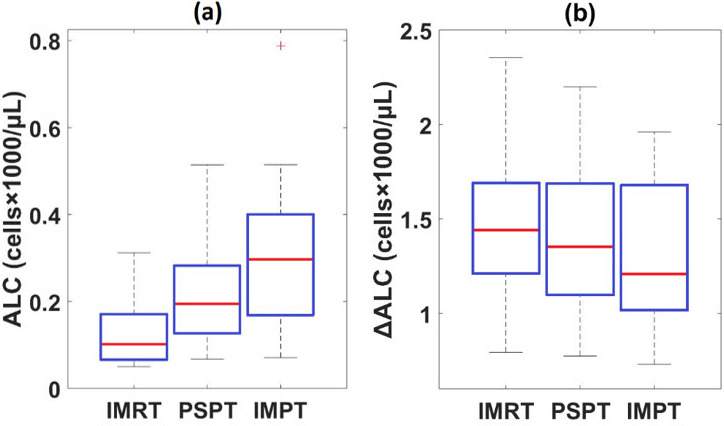
ALC predictions using the exponential fitting method. (A) Box plots indicating estimated ALC after the 3 treatment modalities averaged over 10 esophageal cancer patients. (B) Box plots indicating predicted ALC changes for the same cohort. Abbreviation: ALC, absolute lymphocyte counts.

Weekly measurements of ALC and the total delivered dose for PSPT were used to fit an exponential curve (equation 6 above) for each patient. **Figure 3A** shows box plots of predicted posttreatment ALC values for all patients using this approach for all 3 modalities, and **Figure 3B** shows box plots of predicted ALC changes before and after treatment (ΔALC). Consistent with the predictions of the piecewise linear function approach, the predicted final ALC was the lowest for IMRT; IMPT was estimated to result in a higher ALC than PSPT. The fitted exponential curve and data points used for fitting for all patients are shown in **Supplemental Figure S4**. The average estimated ALCs after treatment, calculated using exponential fitting, were 0.14 (95% CI [0.08, 0.19]), 0.22 (95% CI [0.14, 0.30]), and 0.33 K/μL (95% CI [0.19, 0.45]) for IMRT, PSPT, and IMPT plans, respectively, which shows the same trend as the previous approach.

**Figure 4.**
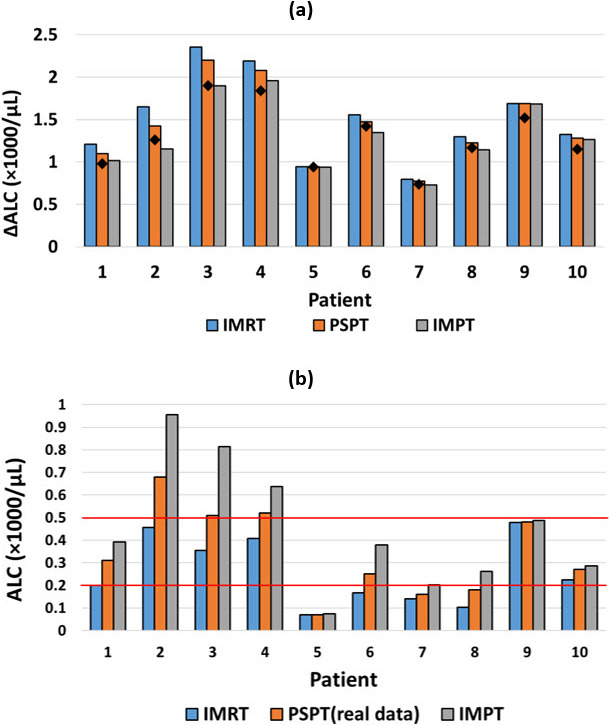
(A) Comparison of predicted ALC changes (baseline − nadir) using the exponential fitting method for IMRT, PSPT, and IMPT plans for 10 esophageal cancer patients. The changes in ALC from measured ALC data for PSPT treatments are indicated by the black diamonds. (B) Predicted ALC nadirs for IMRT and IMPT treatments (blue and gray) using the exponential fitting method compared with the measured ALC nadirs for PSPT treatments (orange). Abbreviations: ALC, absolute lymphocyte counts; IMPT, intensity-modulated proton therapy; IMRT, intensity-modulated radiation therapy; PSPT, passive scattering proton therapy.

A comparison of the predicted ALC change (baseline − nadir) using the exponential model is shown in **Figure 4A**. The ΔALC values for IMRT were higher than those for IMPT and PSPT for all 10 patients. Estimated ALCs had a mean absolute error of 0.125 and a mean squared error of 0.023, higher than for the piecewise linear method.

**Figure 4B** shows the predicted ALC nadirs for IMRT and IMPT treatments, similar to **Figure 2B**. For example, patients 7 and 8 may have avoided grade 4 lymphopenia if treated with IMPT, and patients 1 and 6 had grade 3 lymphopenia with PSPT but might have had grade 4 if treated with IMRT.

**Figure 5.**
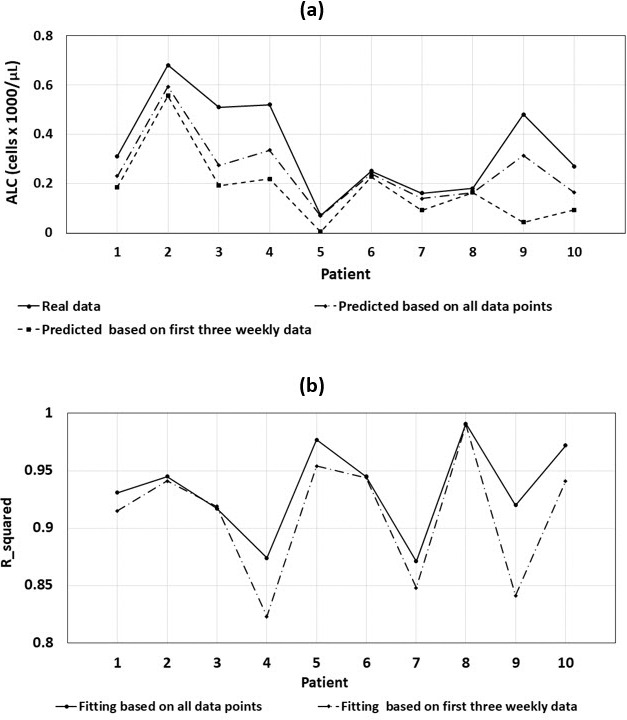
(A) Measured ALC nadirs and estimated posttreatment ALC based on the fitted exponential model using the first 3 weeks' data and all weekly data from PSPT treatments for 10 patients. (B) R^2^ comparison between 2 exponential fittings. Abbreviations: ALC, absolute lymphocyte counts; PSPT, passive scattering proton therapy.

To predict lymphopenia in the early stages of treatment, we used the ALC-dose data for the first 3 weeks to estimate the parameters of the exponential ALC function. The fitted exponential curves using 3-week data for all patients are shown in **Supplemental Figure S5**. **Figure 5A** illustrates the measured ALC nadirs and the estimated final ALC based on 3-week and all-week data after PSPT treatment. ALC predictions based on 3-week data were lower than ALC predictions using all weekly data. **Figure 5B** shows an *R*^2^ comparison between 2 exponential fittings, which was higher for the first exponential fitting for all 10 patients. The average *R*^2^ values for exponential fitting using all weekly data and 3-week data were 93.5% and 90.0%, respectively.

## Discussion

The present work suggests that IMPT would lead to less lymphocyte depletion than PSPT and that IMRT may produce the most lymphocyte depletion. This was demonstrated in both model validation study and treatment planning study. The 2 models of lymphocyte survival performed well in predicting the trend of ALC changes during RT treatment. It is worth noting that this study focuses on comparing RIL risks from different treatments for the same individual patient, rather than associating RIL with a specific treatment for prospective patients and analyzing a large cohort of patients. Studies like this can generate hypotheses for clinical trials for investigating the RIL risks associate with different radiation modalities and competing treatment plans. Although simple models, such as those used in this study, are entirely based on radiation dose and their accuracy in predicting lymphocyte changes may be limited, other recent studies have shown that these models (eg, effective dose to the circulating immune cells), could be clinically useful to evaluate susceptibility to severe lymphopenia for esophageal cancer patients [29, 30].

In particular, the limitation of the piecewise linear model is owing to the assumption that lymphocyte distribution throughout the treatment field is homogeneous. This is not the case in which organs (eg, spleen in the upper abdomen) may have a concentration of lymphocytes, and may have a greater influence on lymphocyte depletion than the body as a whole. Several studies have investigated blood flow models to estimate the dose to the circulating blood during treatment for some cancer sites, such as the lung and brain [31, 32]. These models could be used to determine more precise dose to circulating lymphocytes as an input to piecewise linear model to better understand radiation-induced lymphopenia in future studies. Nevertheless, it is reassuring that our simple model has a good predictive power and potential clinical usefulness.

The exponential lymphocyte survival model built from fitting measured ALC data was also able to predict ALC depletion during treatment. However, it was less accurate than the piecewise linear model for predicting the ALC nadir. For example, **Supplemental Figure S6** shows the comparison of measured and predicted ALC nadirs based on the linear and exponential methods for all PSPT patients. Note that predictions were calculated for 28 fractions (5.6 weeks), but the measured nadirs only were from 4 to 6 weeks among patients. Two possible causes may be (1) the uncertainty in ALC measurements, especially near the end of the treatment course, when the ALC values can be very low, as a result of the rounding errors (ALC measurements in clinical laboratory reports are rounded to the nearest 100 cells/μL); and (2) the variability in time between radiation treatment fraction delivery and blood draw for ALC measurement. For example, spurious increases in ALC were seen for patients 2, 4, and 7 (see **Supplemental Figure S4**). To mitigate the sensitivity of this model to uncertain ALC data, it is crucial to ensure the accuracy and consistency of ALC measurement practice in future studies. In addition, to improve the predictive power, we will investigate approaches to incorporate preclinical predictors into the model in our future work, such as patient age and body mass index [33].

Although it is not clear that the exponential fitting approach will be of benefit in predicting ALC for new patients, as it requires large data sets, this approach is useful in comparative studies of different dose patterns for individual patients, such as in the present work. In addition, a recent study by Ellsworth et al [26] showed that ALC loss kinetics early in the course of treatment are strongly correlated with the extent of ALC depletion during radiation therapy and can be used to identify patients at high risk of developing severe radiation-induced lymphopenia. By only fitting the ALC data in initial weeks of treatment (of the first few fractions), one could estimate of ALC nadir early in treatment course based on the dose distribution and the consideration of individual patients' lymphocyte sensitivity and make midcourse correction with adaptive plans if and when needed.

This study also motivates further studies to investigate the clinical factors that affect RIL risk of different radiation modalities. With help of research on continuing better understanding of lymphocyte distribution throughout the treatment field, radiation dose could be optimized accordingly to avoid lymphocyte killing. For example, IMPT and IMRT plans in this study were optimized using the same conventional dosimetric criteria as the PSPT plans. Additional immune sparing could be possible by optimizing plans with constraints on dose received by volumes of the body (and immune organs at risk, such as the spleen, heart, etc.), which is most promising for IMPT due to its high complexity and flexibility in modulation. Such methods to enhance the ability of IMPT to minimize lymphopenia risk but without compromising tumor coverage and other normal tissues at risk will be studied in our future work.

## Conclusions

This treatment planning study assessed RIL risk and the impact of different dose distributions of IMRT, PSPT, and IMPT on ALCs for 10 esophageal cancer patients. Two methods were proposed to estimate posttreatment ALC, and their reliability in predicting ALC were validated in separate patient groups for each treatment modality. Results from both approaches showed significant lymphocyte reduction associated with treatment. Proton plans showed a lower risk of lymphopenia after the treatment course than did photon plans, and IMPT plans outperformed PSPT plans in terms of lymphocyte preservation.
