# Supplementary material for: Radiation-Induced Lymphopenia Risks of Photon Versus Proton Therapy for Esophageal Cancer Patients
Source: Int J Part Ther. 2021 Apr 7;8(2):17–27. doi: 10.14338/IJPT-20-00086 (PMC8489492; doi:10.14338/IJPT-20-00086)
Supplement: Supplementary file 1 [file ijpt-08-02-03_s01.docx]

**Supplementary Material**


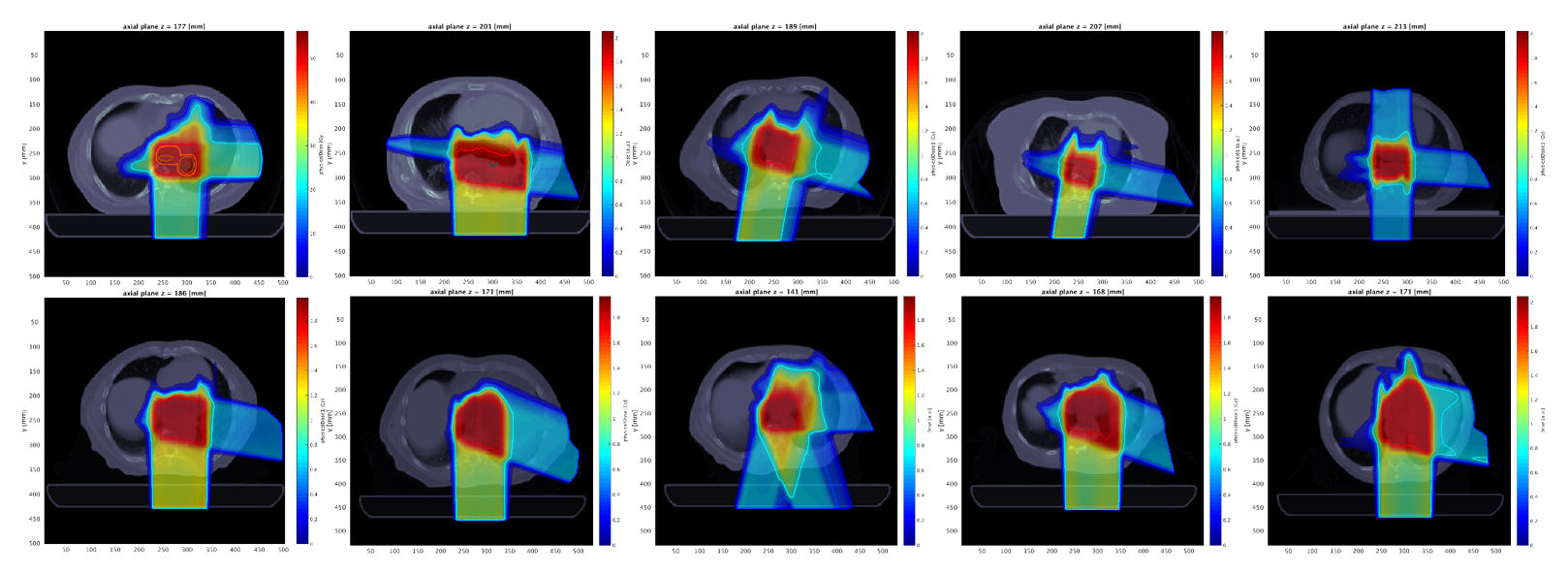


Figure S1. Dose distributions on an axial plane of PSPT plans for 10 esophageal cancer patients.


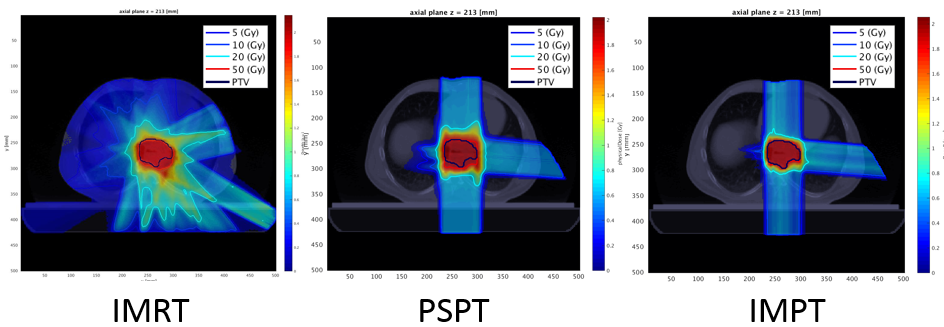


Figure S2. Dose distributions on an axial plane of IMRT, PSPT, and IMPT plans on an axial plane for Patient 5.


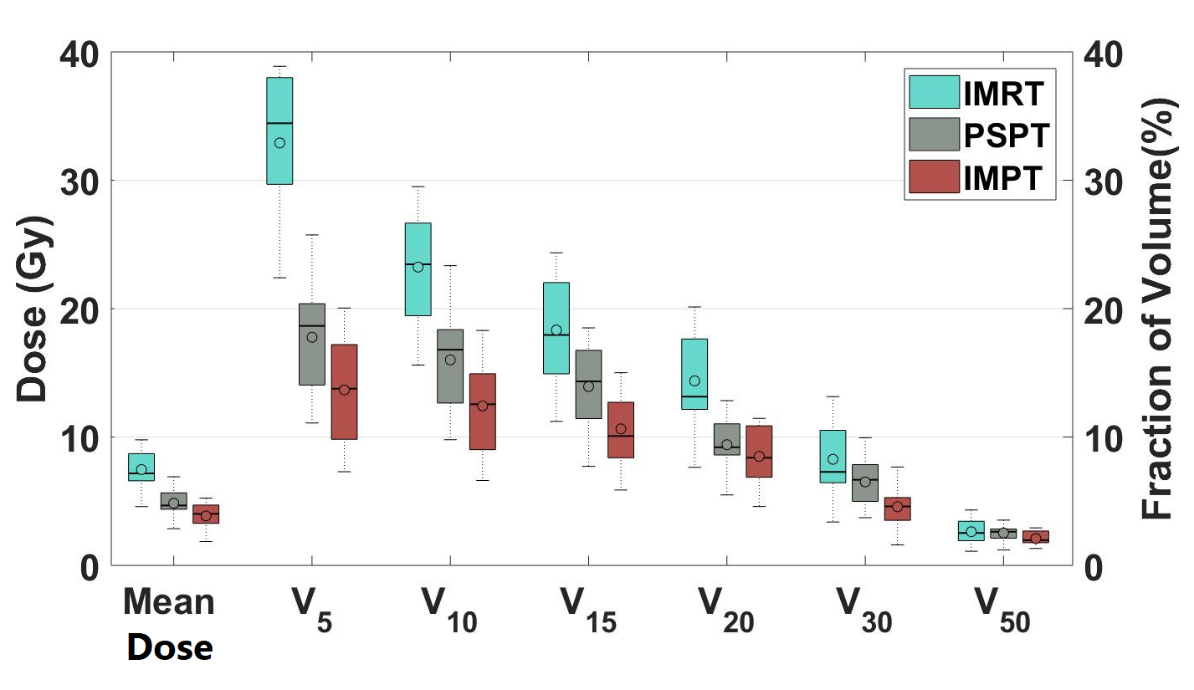


Figure S3. Box plots illustrating different dose-volume indices for the total irradiated volume for 3 treatment plans (IMRT, PSPT, and IMPT) in 10 patients. V_5_ is the fraction of volume receiving more than 5 Gy dose, likewise for other indices. The circles in the boxes indicate the mean value among the 10 patients.


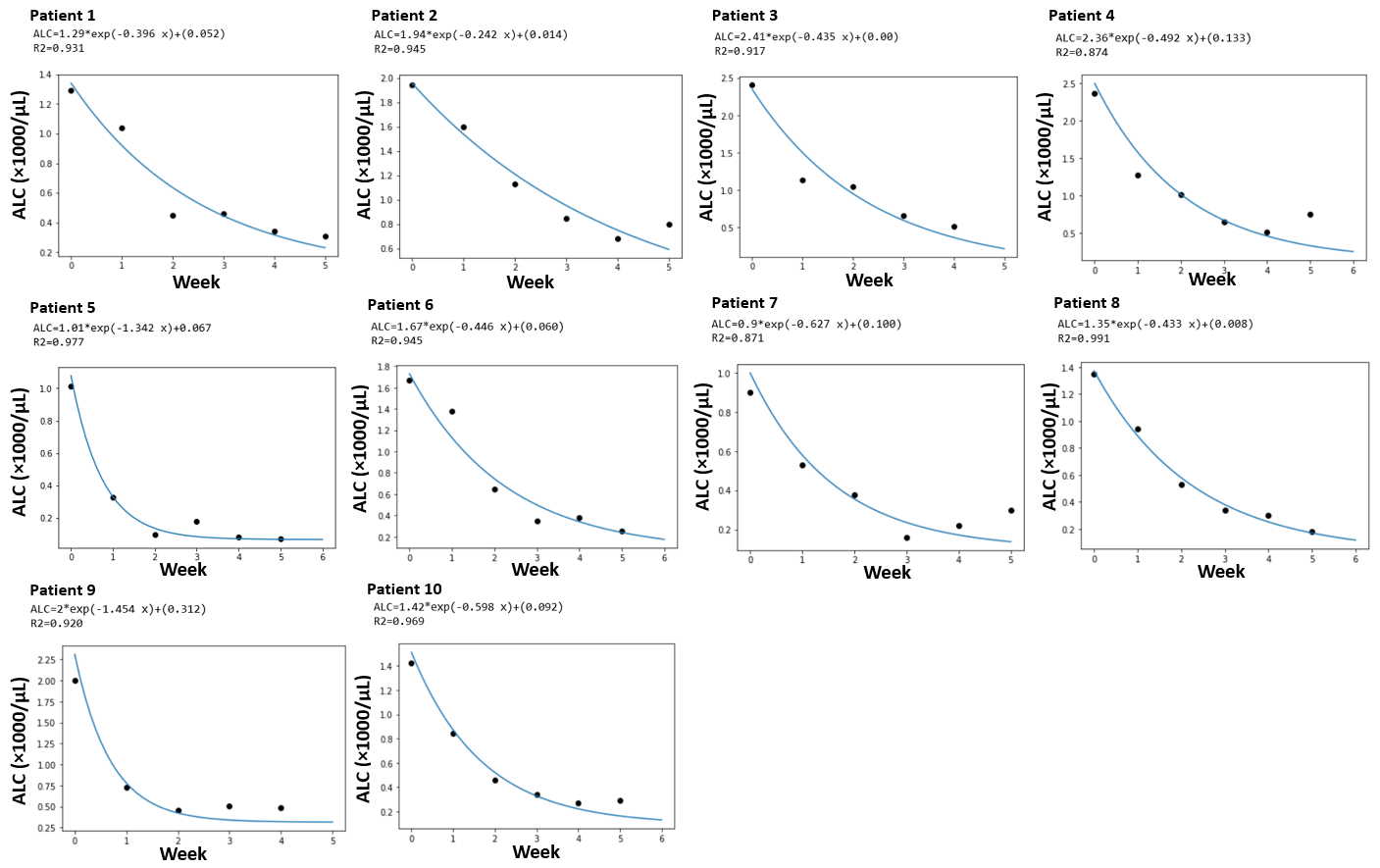


Figure S4. The exponential curves fitted with measured weekly ALC data for 10 esophageal cancer patients treated with PSPT.


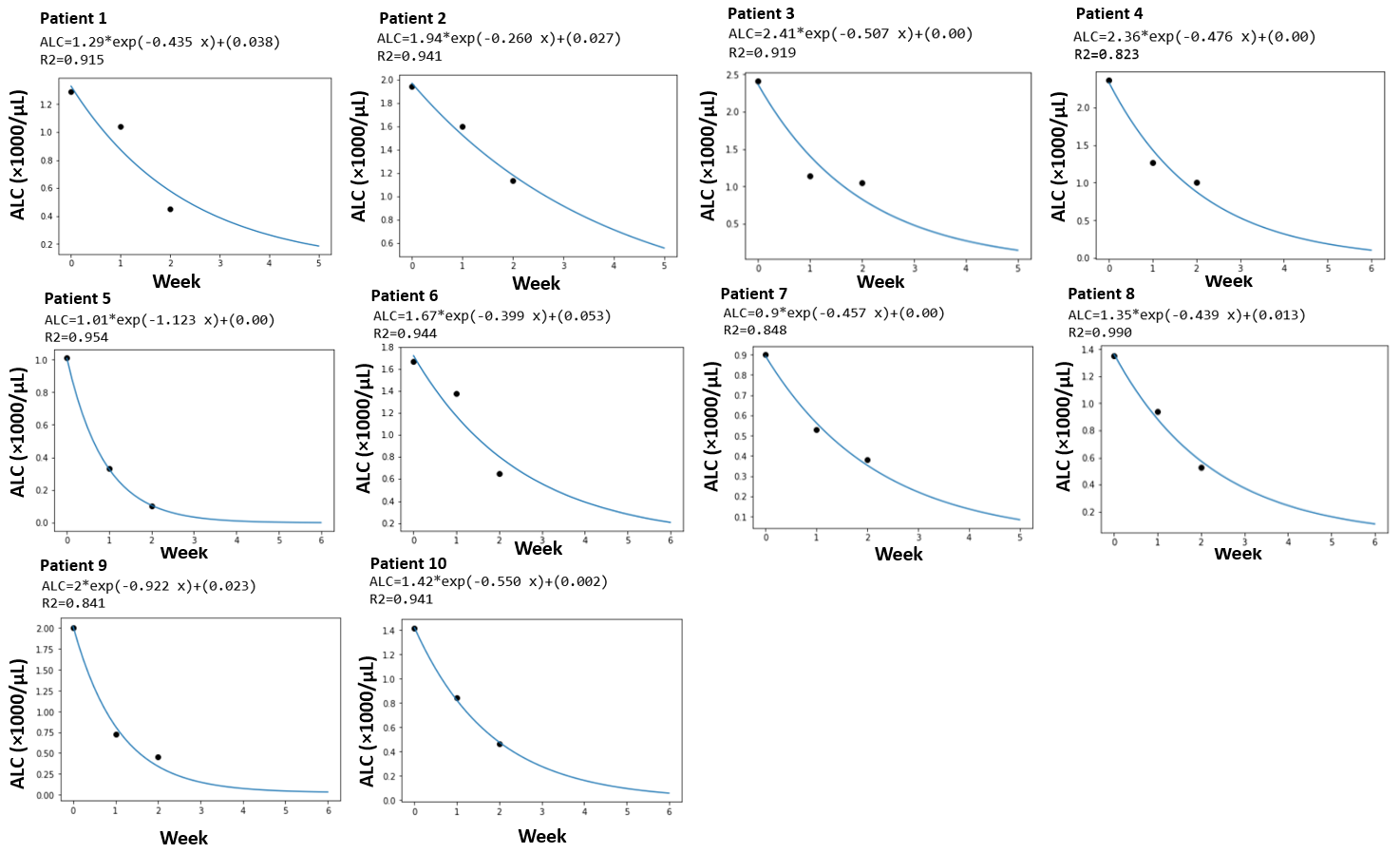


Figure S5. The fitted exponential curve based on the first 3 weeks’ measured ALC data for 10 esophageal cancer patients treated with PSPT.


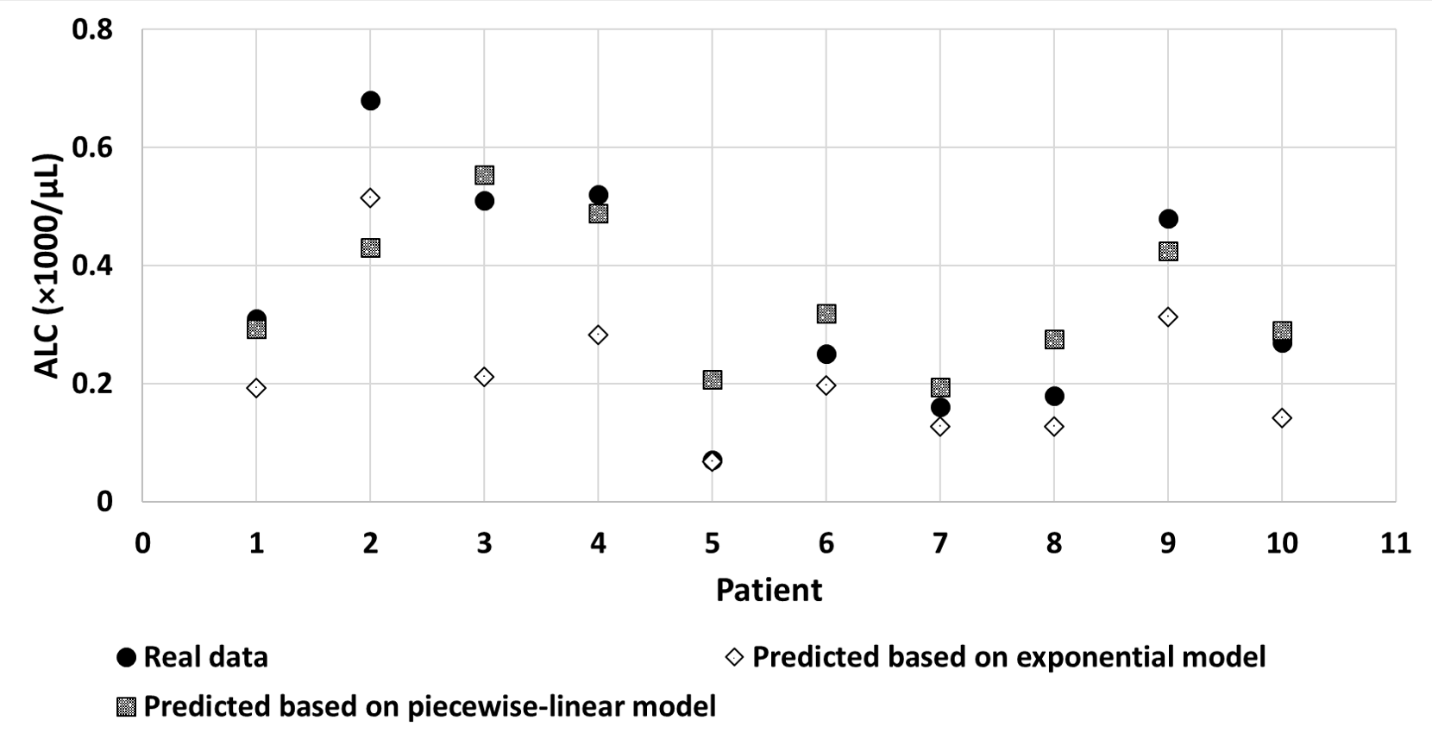


Figure S6. Measured ALC nadirs and the estimated posttreatment ALC based on fitted exponential model and piecewise-linear model for PSPT treatments.

Table S1. ∆ALC (baseline - nadir) based on real and predicted ALC values for patients treated with IMRT, PSPT, and IMPT. ALC values are in unit of cells × 1000/μL and presented by mean ± standard deviation. (MSE: mean squared error; MAE: mean absolute error)

| **RT Modality** | **Real**  $\boldsymbol{\Delta}\boldsymbol{ALC}$ | **Piecewise-linear Model** | | | **Exponential Model** | | |
| --- | --- | --- | --- | --- | --- | --- | --- |
|  |  | **Predicted** $\boldsymbol{\Delta}\boldsymbol{ALC}$ | **MSE** | **MAE** | **Predicted** $\boldsymbol{\Delta}\boldsymbol{ALC}$ | **MSE** | **MAE** |
| **IMRT** | 1.25 ± 0.44 | 1.27 ± 0.43 | 0.005 | 0.064 | 1.30 ± 0.46 | 0.005 | 0.053 |
| **PSPT** | 1.08± 0.52 | 1.09 ± 0.52 | 0.023 | 0.104 | 1.11 ± 0.54 | 0.005 | 0.057 |
| **IMPT** | 0.97 ± 0.58 | 0.98 ± 0.60 | 0.003 | 0.040 | 0.99± 0.59 | 0.004 | 0.058 |

Table S2. Normalized ∆ALC (baseline - nadir) based on real and predicted ALC values for patients treated with IMRT, PSPT, and IMPT. ALC values are in unit of cells × 1000/μL and presented by mean ± standard deviation. (MSE: mean squared error; MAE: mean absolute error)

| **RT Modality** | **Real**  ${\boldsymbol{\Delta}\boldsymbol{ALC/ALC}}_{\boldsymbol{0}}$ | **Piecewise-linear Model** | | | **Exponential Model** | | |
| --- | --- | --- | --- | --- | --- | --- | --- |
|  |  | **Predicted** ${\boldsymbol{\Delta}\boldsymbol{ALC/ALC}}_{\boldsymbol{0}}$ | **MSE** | **MAE** | **Predicted** ${\boldsymbol{\Delta}\boldsymbol{ALC/ALC}}_{\boldsymbol{0}}$ | **MSE** | **MAE** |
| **IMRT** | 0.88 ± 0.06 | 0.89 ± 0.05 | 0.003 | 0.049 | 0.91± 0.05 | 0.002 | 0.038 |
| **PSPT** | 0.75 ± 0.09 | 0.76 ± 0.09 | 0.007 | 0.067 | 0.78± 0.09 | 0.003 | 0.043 |
| **IMPT** | 0.65 ± 0.35 | 0.66 ± 0.35 | 0.001 | 0.027 | 0.66± 0.36 | 0.002 | 0.040 |
